# Real-time motion-enabling positron emission tomography of the brain of upright ambulatory humans

**DOI:** 10.1038/s43856-024-00547-2

**Published:** 2024-06-13

**Authors:** Nanda K. Siva, Christopher Bauer, Colson Glover, Alexander Stolin, Sonia Chandi, Helen Melnick, Gary Marano, Benjamin Parker, MaryBeth Mandich, James W. Lewis, Jinyi Qi, Si Gao, Kaylee Nott, Stan Majewski, Julie A. Brefczynski-Lewis

**Affiliations:** 1 Department of Neuroscience, West Virginia University, P.O. Box 9303, Morgantown, WV USA; 2 University of Kentucky College of Medicine, Lexington, KY USA; 3 Department of Biomedical Engineering, University of California, Davis, Davis, CA USA

**Keywords:** Motor control, Neurological disorders, Social neuroscience, Biophysics, Motivation

## Abstract

**Background:**

Mobile upright PET devices have the potential to enable previously impossible neuroimaging studies. Currently available options are imagers with deep brain coverage that severely limit head/body movements or imagers with upright/motion enabling properties that are limited to only covering the brain surface.

**Methods:**

In this study, we test the feasibility of an upright, motion-compatible brain imager, our Ambulatory Motion-enabling Positron Emission Tomography (AMPET) helmet prototype, for use as a neuroscience tool by replicating a variant of a published PET/fMRI study of the neurocorrelates of human walking. We validate our AMPET prototype by conducting a walking movement paradigm to determine motion tolerance and assess for appropriate task related activity in motor-related brain regions. Human participants (*n* = 11 patients) performed a walking-in-place task with simultaneous AMPET imaging, receiving a bolus delivery of F^18^-Fluorodeoxyglucose.

**Results:**

Here we validate three pre-determined measure criteria, including brain alignment motion artifact of less than <2 mm and functional neuroimaging outcomes consistent with existing walking movement literature.

**Conclusions:**

The study extends the potential and utility for use of mobile, upright, and motion-tolerant neuroimaging devices in real-world, ecologically-valid paradigms. Our approach accounts for the real-world logistics of an actual human participant study and can be used to inform experimental physicists, engineers and imaging instrumentation developers undertaking similar future studies. The technical advances described herein help set new priorities for facilitating future neuroimaging devices and research of the human brain in health and disease.

## Plain language summary

Brain imaging plays an important role in understanding how the human brain functions in both health and disease. However, traditional brain scanners often require people to remain still, limiting the study of the brain in motion, and excluding people who cannot remain still. To overcome this, our team developed an imager that moves with a person’s head, which uses a suspended ring of lightweight detectors that fit to the head. Using our imager, we were able to obtain clear brain images of people walking in place that showed the expected brain activity patterns during walking. Further development of our imager could enable it to be used to better understand real-world brain function and behavior, enabling enhanced knowledge and treatment of neurological conditions.

## Abstract (web summary)

Siva, Bauer et al. use a motion-compatible brain imager to detect task-related brain activity in human participants walking in place. The feasibility of positron emission tomography (PET) neuroimaging of people performing motor tasks or who cannot remain still, is demonstrated.

## Introduction

Current human brain imaging methods are limited by not allowing both natural upright motion and deep brain coverage. This barrier is predominantly attributed to extreme motion sensitivity, exclusively supine brain imaging capabilities of standard horizontal bore scanners, and requirement of special dedicated scanning rooms (e.g., traditional Magnetencephalography (MEG), functional Magnetic Resonance Imaging (fMRI), standard Positron Emission Tomography (PET), and Single Photon Emission Computed Tomography (SPECT))^1^. The neuroimaging systems that do allow for upright motion in real-world environments (Electroencephalography (EEG), functional Near-InfraRed Spectroscopy (fNIRS) and High-density Diffuse Optical Tomography (HD-DOT)) are limited by their reduced resolution or inability to image deeper lying brain regions such as basal nuclei (ganglia), hippocampus, and thalamus^2–4^. These limitations adversely affect clinical and research neuroimaging in terms of the selection of patients, the feasibility of real-world, ecologically-valid paradigms, and the range of behaviorally-relevant tasks that can be performed, such as studies of speaking, social interaction, expressing emotions, and engaging in upright tasks.

First, current imagers capable of both surface and deeper brain structures present limitations of motion tolerance that makes it difficult, if not impossible, to scan persons who cannot remain still, creating major obstacles for research in children, cognitively challenged individuals, and those with disorders such as Parkinson’s Disease (PD), dementias, Autism Spectrum Disorder (ASD), or epilepsy^5^. With PD, for example, because of their involuntary movements, only inferences can be made in researching the progression of medication regimens and pharmaceutical alterations during stages of disease progression where motor control impairments may prohibit unsedated neuroimaging. A portable imaging devise that could measure brain activity in a comfortable, upright position without unnatural movement-controlling interventions would greatly advance research in clinically relevant populations, and thus this remains a major goal in various research fields of neuroimaging.

Lack of motion tolerance also affects the type of tasks a participant can perform, and there is a need in neuroscience to have paradigms be as ecologically-valid as possible; i.e., to obtain the most accurate neural correlates of a social, emotional or physical task, participants need to be able to be upright when relevant, and react with postural changes, physical and emotional expressions, and verbal responses. Due to current motion limitations, researchers must employ compromises to ensure feasibility, while creating often impoverished representations of the real-world. Most commonly, behavioral paradigms entail viewing a small field of view (FOV) screen and/or hearing through headphones^6,7^, using imagery instead of behavior, such as for studying non-physical balance training^8^ (imagining one is balancing while lying in scanner), in-room hand-holding^9^, hyperscanning two persons while they socially interact simultaneously in linked scanners^10^, and the use of MRI-compatible custom built horizontal cycling^11^ and stepping apparatuses^12^. Participants must also remain unnaturally still during traditional neuroimaging, an extreme example of which was a study of humor wherein participants were trained to suppress physical laughter prior to the MRI session^13^. Thus, limitations to movement and unnaturalness of task and behavior, continue to be a major barrier to studying the brain-based mechanisms of behaviors as they would be in the real world.

In contrast, for motion-tolerant imagers, brain activity information is diminished or missing from brain regions like basal nuclei, hippocampus, and limbic regions critically involved in motor, balance, social, and emotional tasks. HD-DOT and fNIRS technologies have allowed for natural movement and behaviors such as walking^14^, eating behaviors^15^, and social interaction^16^. However, the optical light mechanisms of these modalities only penetrate a short distance into the brain, leaving unseen the activity in deep brain structures known to be critical for balance, reward, addiction, memory, and other clinically-relevant functions affected by neurological injuries or diseases^17,18^. More recently, developments in OPM-MEG allow deeper brain coverage along with a small degree of head and upper body movements, enabling more natural tasks, such as playing a custom non-metal musical instrument^19^. However, restrictions still remain, as large movements like walking cannot be accommodated plus OPM-MEG imaging requires a special Radio Frequency (RF) shielded room, a bespoke helmet, metal restrictions, and there is reduced sensitivity in deep brain structures such as the thalamus, basal nuclei, and hippocampus^19^. Overall, these developments demonstrate the acute need for developing neuroimaging tools that can accommodate robust motion, and provide functional measures of deep brain structures^20^.

Enabling PET imaging to become an upright and motion-tolerant imaging modality would have a special advantage, as PET has the unique ability to quantitatively measure the distribution and intensity of metabolite uptake and neurotransmitter upregulation in the brain (e.g., glucose, oxygen, neuroreceptor agonists, and antagonists). This makes advances in ambulatory PET technology appealing for monitoring pharmaceutical alterations in the brain, while enabling research with moving participant populations and ecologically-valid paradigms. Whole brain PET imaging paradigm adaptations have allowed for human behavioral tasks that require motion, including repeated O^15^-H_2_O injections^21^, but are limited by the higher activity exposure of this ligand and requirements of having a very close-by cyclotron^22,23^. Next, Delayed-PET studies inject research participants outside of the PET scanner just before performing a task that cannot be performed in the scanner, with metabolic markers like Fluorodeoxyglucose (F^18^-FDG) or neurotransmitter specific ligands like C^11^-raclopride (a neuroreceptor agonist for dopamine D2 receptors), with the ligand uptake changes occurring during the task. Delayed-PET can obtain post-activity imaging of complex natural tasks such as driving^24^, freezing of gait in PD^25^, social interactions with speech/gestural/physical reactions^26^ and emotional responses to music^27^; however, the image is more like a long-exposure photograph of the 10 to 20 min activity period, and requires a separate-day baseline scan with an extra radioligand dose. An ideal PET paradigm would retain quantitative imaging benefits but allow same-session collection of multiple tasks and baseline, all while remaining upright and allowing natural motion.

In order to fully utilize a real-time motion-tolerant PET imager, one must take advantage of ligand delivery methods that enable multiple task/baseline periods such as multiple bolus^28^ and bolus/infusion (bolus followed by steady-state infusion) techniques^29–32^^,^. Bolus-infusion F^18^-FDG studies in humans using traditional PET scanners have demonstrated task appropriate activity for visual and auditory task paradigms, with a same-session baseline control, and with ON/OFF task period cycles as short as one to two minutes^30–33^. In terms of physical device changes, the massive heavy detectors that limited PETs motion tolerance can be replaced with lightweight detectors due to advances in detector materials, such as solid-state Silicon Photomultiplier (SiPM) technology^34,35^, which have been used in head-dedicated brain scanners^36,37^, although these so far are fixed and do not tolerate head motion^38^. One of the first SiPM PET imagers that was constructed to allow for large natural movements was the RatCap, designed for small animals^28,39^. This RatCap design thus served as a primary inspiration for the creation of our first prototype imager for human use.

Our group previously developed a wearable PET helmet prototype (termed Helmet-PET), wherein patient-participants were imaged while seated still and then while rotating their head slowly from side to side, demonstrating a general resting-state pattern image comparable to that obtained in their same-day clinical PET scan^40^. We subsequently modified that prototype to enable a greater range of motion while the wearer could be in a standing position and could move about in place or on a treadmill. This modified prototype (termed Ambulatory, Motion-enabling PET, or AMPET), is a lightweight (~3 kg) imager supported from above by a balanced bungee support that utilizes a comfortable friction fit to the head, similar to wearing a hard hat.

In the present study, we sought to test our AMPET prototype in volunteer participants by challenging the helmet device with more robust motor tasks, aiming to replicate a variant of a delayed PET/fMRI block paradigm study that included walking in place^41^. We performed F^18^-FDG AMPET scanning while a convenience sample of patient-participants (cancer patients who were already scheduled for a clinical PET scan) performed the task of walking in place (walking-in-place) relative to standing still (standing-at-rest). The participants were imaged and began performing the task immediately after a pre-dose bolus of F^18^-FDG ligand that was within their allowed dose for the day. We selected three criteria goals for validating the proof-of-concept of the AMPET prototype’s motion tolerance and neuroimaging capabilities. These three validation measures included: Validation #1 of no or limited motion artifacts during robust motion during the walking-in-place versus standing-at-rest task, Validation #2 of differential activation of various a priori defined cortical regions of interest (ROIs) previously reported to be related to leg movement tasks from a previous Delayed-PET study^41^, and Validation #3 of differential activation to walking movements in deep brain structures, such as the thalamus and basal nuclei, that are normally compromised in motion-tolerant surface imagers.

Since our three primary goals were simply to determine AMPET prototype’s feasibility for purposes of prioritizing future advances for upright neuroimaging, we decided to reduce potential burden on this vulnerable population of cancer scan patient-participants by not acquiring arterial blood sampling for quantitative imaging, as is done for traditional PET research imaging. This compromise, and not taking advantage of the bolus-infusion ligand delivery, created a relative measure of a priori brain region activation with a long-exposure photograph effect similar to Delayed PET. This still allowed for motor artifact calculations, and comparisons with a patterns of brain region activation seen in other walking paradigms, each with their own technology-related limitations, as described above. We could thus gain valuable feasibility-related information to inform future studies highlighting state-of-the-art developments for ambulatory movement paradigms using motion-enabling, whole-brain imaging technologies.

We find in this study that all three validation measures are confirmed, such that no significant motion artifact is measured, and task-related activity is observed in a priori expected regions, with the significantly highest activity in bilateral leg representing regions of primary motor cortex, followed by supplementary motor control and visual-motor brain regions. These validations are further confirmed with a case-study leg amputee patient showing motor cortex dominance representing the existing leg, as well as a subset of participants showing trend level activation of basal nuclei motor regions.

## Methods

### Participants

West Virginia University’s Institutional Review Board (WVU IRB) approved this study as device testing, i.e., not having a neuroscience discovery related to brain function as the goal. The study did not meet the IRB definition of a clinical trial and was considered observational. Ease for participants was emphasized, so it involved no arterial sampling and used bolus injection. The IRB approved a sample size of 10–12 participants, a number chosen to minimize patient burden whilst giving a range of patient experiences. Participants were all white, representing the West Virginia demographics (7 females and 4 males), with a mean weight of 76.1 kilograms (range 40.4–112.0 kg), and mean age of 53 years (age 25–66). All ethical regulations were followed, per WVU Office of Human Research Protections policy, and written informed consent was obtained from participants. The inclusion and exclusion criteria were that any consenting participant had to be over 18 years old and that participants were required to already be scheduled for a clinical PET scan for non-brain cancer on the same day, and they were recruited during the phone reminder of the clinical scan. Participants were required to have the ability to walk in place for up to 10 min, without difficulty or balance problems. Exclusion criteria included issues with upper spine, and other head/neck pain or mobility issues.

One consented participant’s data was omitted from the analysis due to an injection amount below the allotted range of 1–2 millicurie due to a timing delay. For the walking task analyses, two participants’ trials were omitted due to AMPET helmet placement that was inadvertently positioned below the leg motor Regions of Interest (ROIs). Thus, we had a sample size of *n* = 10 for the motion tolerance analyses (Validation measure #1), and a sample size of *n* = 8 for the walking-in-place task analyses (Validation measure #2), and a subset of *n* = 4, *n* = 5 (of the 10) participants for re-placement of the AMPET to measure deep brain structure activity and M1-reliability (Validation measure #3). One participant was not in range of either full primary motor cortex (M1) coverage or of the deep brain structure coverage after re-placement.

#### Case study participant

We did not specifically recruit an amputee participant. One participant, recruited in the same way as the other participants, fully met all inclusion/exclusion criteria specified for the study. In terms of task performance, this included being able to walk and stand unassisted. Although they mentioned having a right leg prosthesis (hip to foot amputation, non-articulating prosthetic leg), since they were fully comfortable with the task, this did not violate our criteria. This allowed for collection of data pertaining to having only one leg in motion. Questions such as the duration of the prosthetic usage in this patient-participant was not asked due to IRB protocol regulations.

### AMPET imager specifications

The AMPET imager prototype used in this study consisted of a ring of 12 pixelated radiation detector modules, each covering an active detection field of 48 × 48 mm. Detector modules were comprised of arrays of 3 mm multi-pixel photon counters (MPPCs) solid state silicon photomultipliers (SiPMs) from Hamamatsu Photonics (Hamamatsu City, Japan) coupled to 1.5 × 1.5 × 10 mm lutetium-yttrium oxyorthosilicate (LYSO) pixelated scintillation crystal arrays. The imager ring had a Field of View (FOV) of 21 cm, spatial resolution at the center of detector was 2 mm Full Width Half Max (FWHM) in the tangential direction and 2.8 mm in the radial direction, and weight of 3 kg. Although AMPET helmet could be supported on the head of most healthy people at rest, in our study we opted to suspend it from a flexible cord attached to a single point support system so that the patient-participants would not feel the weight of the helmet while moving their head. For the walking-related tasks (see below) the support enabled a comfortable range of limited 3D head motions typical of walking behavior. Other technical aspects of the suspension apparatus, also used with the previous Helmet_PET prototype, are described in more detail in ref. ^40^. The Helmet_PET imager, now reconfigured as the AMPET prototype, had since been revised to include in-module amplifiers, a temperature monitoring system, and a remote adjustment of SiPM bias voltages. The temperature of each of the 12 detector modules was sampled separately due to temperature-related performance variability; this variability was monitored and mitigated by corrective adjustments to within an event signal energy acceptance window.

### Walking-related tasks

**Fig. 1:**
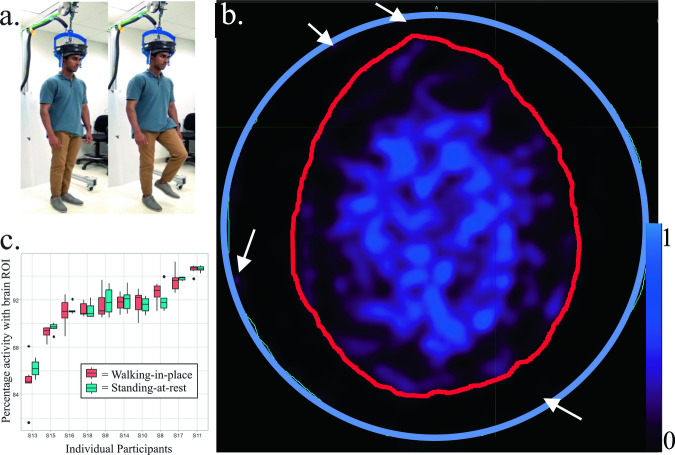
AMPET set-up and motion artifact analyses. The AMPET helmet set up and motion artifact analyses (*n* = 10). **a** Set-up showing a model (co-author N.S.) demonstrating the task of standing-at-rest and then walking-in-place. **b** Example of Motion Tolerance ROI’s drawn in an axial brain image by tracing skull (red outline) versus imager inner edge marking the FOV (blue circle). White arrows point out a few small regions of activity outside the brain that reflect some form of artifact (on average 5.4–14.9% of total, depending on anatomy of participant). The stability of the ratio of activity inside versus outside the brain was used in the motion variance-related measurement differences in walking-in-place versus standing-at-rest, with blues and purples indicating activity. Scale bar indicates relative uptake signal intensity. **c** Boxplot of ROI average amplitude during active walking-in-place versus standing-at-rest periods. The upper and lower limits of the box are the lower and upper quartiles, the ends the whiskers are maximum and minimum of data. Dots indicate value of the individual participant measurements included in the averages. If the imager moved significantly relative to the brain, walking-in-place versus standing-at-rest would show an altered ratio, due to movement in or out of the axial imaging plane. The results show no significant differences as a group or individually in any participant tested. Source data can be found in Supplementary Data 1.

The AMPET helmet was paced on the participant’s head as they were standing upright, and the friction fit was tightened, with the full weight of the helmet supported by an overhead bungee (see example on co-author model in Fig. 1a). A computer screen with a fixation point and displaced written instructions (walk vs. rest) was placed in front of the participants; they were asked to maintain gaze on the fixation point for the duration of the imaging session. During the first six-minute period (0–6 min following the F^18^-FDG bolus injection), each participant was instructed to perform the upright motor task, wherein they alternated every 30 s between standing still (standing-at-rest) and then walking in place at a natural pace (walking-in-place; average pace of 1.3 steps/s). The block paradigm task began with standing-at-rest for 30 s to minimize and/or address possible temporal equilibration artifacts. Limiting walking time to 30 s intervals was recommended by the physical therapist on our team to ensure that these cancer patients had ample time to rest after the mild exertion from walking-in-place. The alternations also allowed us to test for spatial motion artifacts via activity changes outside the brain during the walking-in-place head movements versus standing-at-rest for Validation measure #1. Note, however, that due to the ligand delivery method of bolus injection of sequestered F^18^-FDG without arterial sampling, any task related differences between these periods could only be reported as signal change (i.e., could not be directly quantified).

The following five minutes (minutes 6–11 following the injection) represented a task transition period, and those data samplings were excluded from the analyses. This transition period allowed time for our patient-participants to move to a seated position for the next set of tasks. During this transition, a chair was moved under the hanging imager, and the AMPET helmet was lowered onto the seated participant’s head using a secure pulley system (e.g., see ref. ^40^). Since full uptake had not yet occurred, participants were asked to continue with a walking-in-place leg movement task, in which they were instructed to alternate (30 s each) between sitting still (seated-rest) and then walking leg lifting movements while seated (seated-walking) during minutes 12–18. The AMPET imager position was slowly and safely readjusted onto the participant’s head in one of two configurations: five of the participants were randomly assigned to have the imager placed lower around their head to image deeper lying brain structures, while five participants had the imager continue to capture the top portion of the brain comprising primary motor areas. Five additional minutes of imaging (minutes 11–16 post-injection) data were collected.

### Experimental AMPET scan

Patients were brought into the imaging laboratory and written consent was obtained under WVU IRB guidelines. The AMPET prototype imager was gently fit to the participant’s head using the adjustable flexible fitting mechanism. The imager was positioned by visual inspection to include the top ~4 cm portion of the head. Without more elaborate placement guidance, this provided the best estimation for capturing bilateral leg motor cortex, located in the most superior region of the motor cortex strip defined a priori in an earlier walking-related study using Delayed-PET and MRI measures^41^.

Once properly situated and ready to walk in place, a pre-dose of 1–2 millicuries of F^18^-FDG (of their total prescribed clinical dose of ~10 millicuries, or ~370 megabecquerels (MBqs)) was delivered as a bolus injection by a licensed nuclear medicine technologist. Imaging began immediately following the injection; and the experimental session, including positioning, lasted a total of roughly 25 min. We utilized a simple bolus injection immediately before the walking-related tasks, though with no arterial blood sampling for overall patient comfort (see Introduction). With this approach we could test for brain-misalignment motion artifact presence during walking-in-place versus standing-at-rest period conditions (Validation measures #1 and #2), and during the seated-walking versus seated-rest conditions (Validation measures #1 and #3), by extracting sampled data within the respective periods of time^28^.

### Clinical PET scans

We utilized the clinical Siemens PET and computed tomography (CT) brain scan for AMPET image registration, and also for assessing activity profiles of the a priori selected ROIs during rest. This latter measure was to assess whether there was any unanticipated tonically high activity in any brain regions, thereby serving as a critical baseline control condition. After completing the AMPET prototype scanning, participants were walked to the nearby clinical PET imaging facility with a Siemens mCT PET/CT and administered their remaining dose of F^18^-FDG (~9 millicuries). Then they were placed in a dim-lighted room and asked to relax by sitting still for approximately 60 min so that full FDG uptake in brain was reached. Finally, patients remained relaxed and were instructed to remain still for their scan period, including the 3-min brain scan, which was part of the standard of care protocol for cancer patients at our institution.

### Data collection

The imaging data was collected in a list-mode format with a timestamp for each coincidence event detection of photons radiating from the annihilation of positrons. For this study the time-series of coincidence events were then divided into different task periods (i.e., resting versus walking movement periods), and after imager readjustments.

### Image processing and reconstruction

Tomographic images from the AMPET research imager were created using a custom-developed iterative reconstruction software based on a Maximum-Likelihood Expectation-Maximization (MLEM) algorithm^42^. Reconstruction employed 10 iterations with 2 × 2 x 2 mm^3^ isotropic voxels and attenuation correction in the volume of the brain. Corrections for random coincidences (scatter) were ignored and presumed inconsequential for purposes of our basic validation measures. Uniformity correction was applied using a previously acquired flood correction in which the target images were divided by images produced using a uniformly radioactive (less than 500 microcurie) cylinder encompassing the entire AMPET prototype’s FOV^40^. To avoid any potential non-uniformity effects due to temperature sensitivity of the photodetectors, we utilized a flood correction image using uniform water cylinder, collected at approximately the same temperature as during patient scanning conditions. Once corrected for uniformity, images were viewed and initially processed using ImageJ research software (http://imagej.nih.gov/ij/, Bethesda, Maryland, USA) and blurred with a smoothing algorithm, with a pixel value of the average of the 3 × 3 (nine) pixel region including the 8 cardinal pixel neighbors. The most superior and inferior slices of the brain in all AMPET scans were removed due to expected edge artifacts of the scanner at both axial FOV edges. The resultant images were then converted to Digital Imaging and Communications in Medicine (DICOM) standard files.

Both the AMPET and clinical PET DICOM files from each distinct participant and condition were imported into MIM software (version 6.6) and analyzed using MIMneuro and MIMfusion. First, the clinical PET scans of each participant were aligned to the CT scans using the MIM automated BrainAlign algorithm (MIM Software, Inc., Cleveland, Ohio, USA). Next, the AMPET scans were aligned to PET/CT images using the same algorithm. If necessary, images were manually adjusted to co-register the AMPET images to correct for any relative positional errors of the automatic process, achieved upon visual inspection by adjusting the alignment of the outlines of brain slice images (e.g., cortical gyri and sulci in Fig. 1c) from the AMPET versus clinical PET anatomical scans.

### ROI selection

For motion artifact analyses, the outline of the brain in a participant’s mid-axial slice was traced (e.g., Fig. 1c, red outline) to demarcate activity inside the brain versus outside the brain, the latter including dura, skull, and scalp. For the walking-related task analyses, left and right hemisphere ROIs reported to encompass the leg and foot representations in primary motor cortex (leg-M1) were derived based on the coordinates provided by a previously published PET study of a walking task^41^. The ROIs were created as spheres with 25 mm diameter from the center of mass of the reported coordinates. Bilateral brain ROIs were combined for the main analyses (though see the amputee case study below). The central coordinates for the left and right leg-M1 ROIs were based on that reported in Montreal Neurological Institute (MNI) space (±12, −38, 72): The MIM system, however, had its own template space, and this corresponded with MIM template space of right hemisphere +8, 20, −51; and left hemisphere −8, 20, −51^43^. In addition to these a priori cortical ROIs, several other regions were chosen from the MIM database of 43 healthy age-matched controls that fell within the AMPET prototype’s brain coverage FOV. The bilateral regions included the supplementary motor area (SMA), precentral gyrus (lateral portions of M1; face and upper limb representations), postcentral gyrus (lateral portions of sensory cortex; S1 face and upper limb representations), precuneus (superior aspects), and frontal lobe regions that anatomically fell well outside of cortices traditionally reported to be activated during motor tasks.

### Image normalization

Image intensity values were normalized by using average intensity from a lateral frontal cortex ROI (MIM template coordinates: 25, −15, −45) because coverage of this region was present in all participants and presumed to not be involved in the motor task performances. This allowed relative uptake values (RUVs) of a target brain region to be presented in ratio form by dividing by this normalization region having similar cortical expanse and blood flow properties but different task representation. Use of this baseline further aided with Validation measures #1-3 by enabling indirect comparison of walking-task AMPET pattern of activation with that of resting data from the clinical PET scanner.

### Motion tolerance analysis

To test for artifact during robust motion versus rest, activity within the whole brain and its spill-over to surrounding regions was compared between the walking-in-place and standing-at-rest periods of the upright walking task. In this manner, imaging during walking movements were compared to a baseline standing rest without motion that was specific (normative) for each patient. For this purpose, the initial six-minute post-injection imaging data window was divided into twelve 30 s images, of the alternating rest/walking blocked task periods. We selected a middle slice image (from the first 30-s interval) and manually drew a whole brain ROI (e.g., Fig. 1b, red outline) and an entire FOV ROI (blue outline) for each patient to be used for all twelve 30 s intervals for a given patient-participant. The ratio of whole brain activity to entire FOV total voxel counts and resulting variability was then calculated (Fig. 1c, boxplots, Source data can be found in Supplementary Data 1). A mixed-effects model was applied for statistical analyses, which accounted for the correlation within participants, and assuming independence across individual participants.

To quantify the ratio change of detected coincidence events into measures of distance (of motion-related offset of AMPET relative to the brain), we utilized the phantom measures for calibration. Percentage shift of voxel ratios were calculated, based on the size of a circular phantom similar in size to a brain. This had the necessary assumption that our ratio change was 100% due to motion and not due to outside factors such as uptake across time, and scatter activity from the neck/chest area.

Using the illustrated equation below, areas (A) would be the area of our 10 cm diameter phantom, 314.16, so 0.13 cm distance would yield a 1% change in standard deviation. For example, if a circular flood phantom (radius = 10 cm) was shifted 5 mm away from its original location, the resulting activity would be 96.82% of the originally measure.

1 $$A = 	\, r^{{\wedge }}2* \arccos \left(d{{\_}}1/r\right)-d{{\_}}1* {sqrt}\left(r^{{\wedge }}2-d{{\_}}1^{{\wedge }}2\right) \\ 	+R^{{\wedge }}2* \arccos \left(d{{\_}}2/R\right)-d{{\_}}2* {sqrt}\left(R^{{\wedge }}2-d{{\_}}2^{{\wedge }}2\right)$$

### Statistics and reproducibility for motion data

Statistical analyses were performed using R software (version 3.6.3, The R Foundation for Statistical Computing, Vienna, Austria). Descriptive statistical analyses were performed to summarize the data including means, standard deviations, and boxplots to demonstrate the data distributions. Because the multiple measurements on motion artifacts were collected in both walking-in-place and standing-at-rest conditions for each participant (6 each, 2 ROIs), the dependence between the repeated measurements were adjusted in the data analyses. To assess the difference between walking and still conditions with multiple paired data structure, a mixed effects model was applied, accounting for the dependence on the repeated measurements from the same participants. The fitted model was examined using the standard diagnostic methods including a normality test and Akaike information criterion.

### Statistics and reproducibility for task data

Task data were analyzed using JASP software (JASP Team (2023) version 0.8.1.1) and Microsoft Excel (version 14.6.5). All reported results were statistically significant at least at *p* < 0.05. Data were tested for normality using the Shapiro-Wilk test before applying statistics (all normally distributed). All data were within three times the interquartile range; there were no outliers. In all investigated ROIs, mean intensity (activation) was calculated by taking the combined total voxel value and dividing by the regional volume (in ml).

We first compared the mean activity within the bilateral leg-M1 ROI with the mean activity within the entire brain in the AMPET prototype’s FOV and with the mean activity of five other ROIs (6 comparisons) during the first 6 min of uptake. We also tested if activity in the SMA ROI would be elevated compared to activity in the other four ROIs (4 comparisons) during this time period. Finally, we conducted the same comparisons between the cortical ROIs (6 + 4 comparisons) when participants were imaged with the clinical PET scanner after full injection (~9 millicuries) about an hour after the AMPET scanning. (For thoroughness, we additionally calculated all possible comparisons, but they are not the variables of interest for this study and are thus not included here in the number of comparisons. The values are reported in the Supplementary Table 1a). Two-tailed independent *t*-tests were used to compare activation between ROIs.

After repositioning the AMPET around the head to capture deep lying brain regions in five of the participants, for minutes 12–18, we compared the mean activity within the caudate nucleus with the mean activity of four other ROIs (4 comparisons). We also compared the mean activity within the putamen with the mean activity of three other ROIs, and between the mean activity with the thalamus with the mean activity of the two other ROIs (3 + 2 comparisons). For the other set of participants (*n* = 5) we positioned for continued M1 coverage. In three of these participants, the frontal lobe ROI was covered yet incompletely, but significance of leg motor ROI vs. Frontal Lobe was significant regardless of whether these were included (0.00088 all included vs. 0.0037 only full coverage). For the repositioned leg-ROI participant subset, we are only examining leg motor ROI vs. Frontal lobe (1 comparison).

### Reporting summary

Further information on research design is available in the Nature Portfolio Reporting Summary linked to this article.

## Results

### Motion tolerance

Blurred edges and ghosting effects that are indicative of motion artifacts were not visually detected (see brain image and ROI in Fig. 1b). To explore the potential for artifact due to motion on a smaller scale and determine the validity of examining small brain region ROIs with this imager, we evaluated the level of motion artifacts by comparing the post-baseline whole brain ROI variability within each patient for the walking-in-place versus standing-at-rest periods (Fig. 1c boxplots). Motion of the imager relative to the head would change the ratio of activity during motion periods versus rest periods, since the high activity inside the brain vs outside the brain would be moving in and out of the brain-defined region of interest. Activity outside the brain is normally low, although affected by activity in the dura, fat, scalp, and skull, by scatter from other sources in the patient’s body, and by gaps in the detector with artifact not canceled by the flood subtraction. These factors should remain constant for a given participant if the scanner is moving precisely with the head, although minor influences such as the overall brain uptake or scatter from greater muscle activity in walking might occur. We applied a mixed effects model analysis which accounted for the correlation within participants and the independence of individual participants. This fitted mixed-effect model analysis showed no significant differences between the two ratios (inside vs. outside brain for walking-in-place and inside vs. outside brain for standing-at-rest blocks, *p* = 0.25). In terms of estimated motion shift magnitude, we calculated that these AMPET-to-brain misalignment shifts, if the activity differences are mostly attributable to motion as described above, would average 1.3 mm (see Methods, Motion tolerance for details). These outcomes supported Validation measure #1.

### Task-related activity

**Fig. 2:**
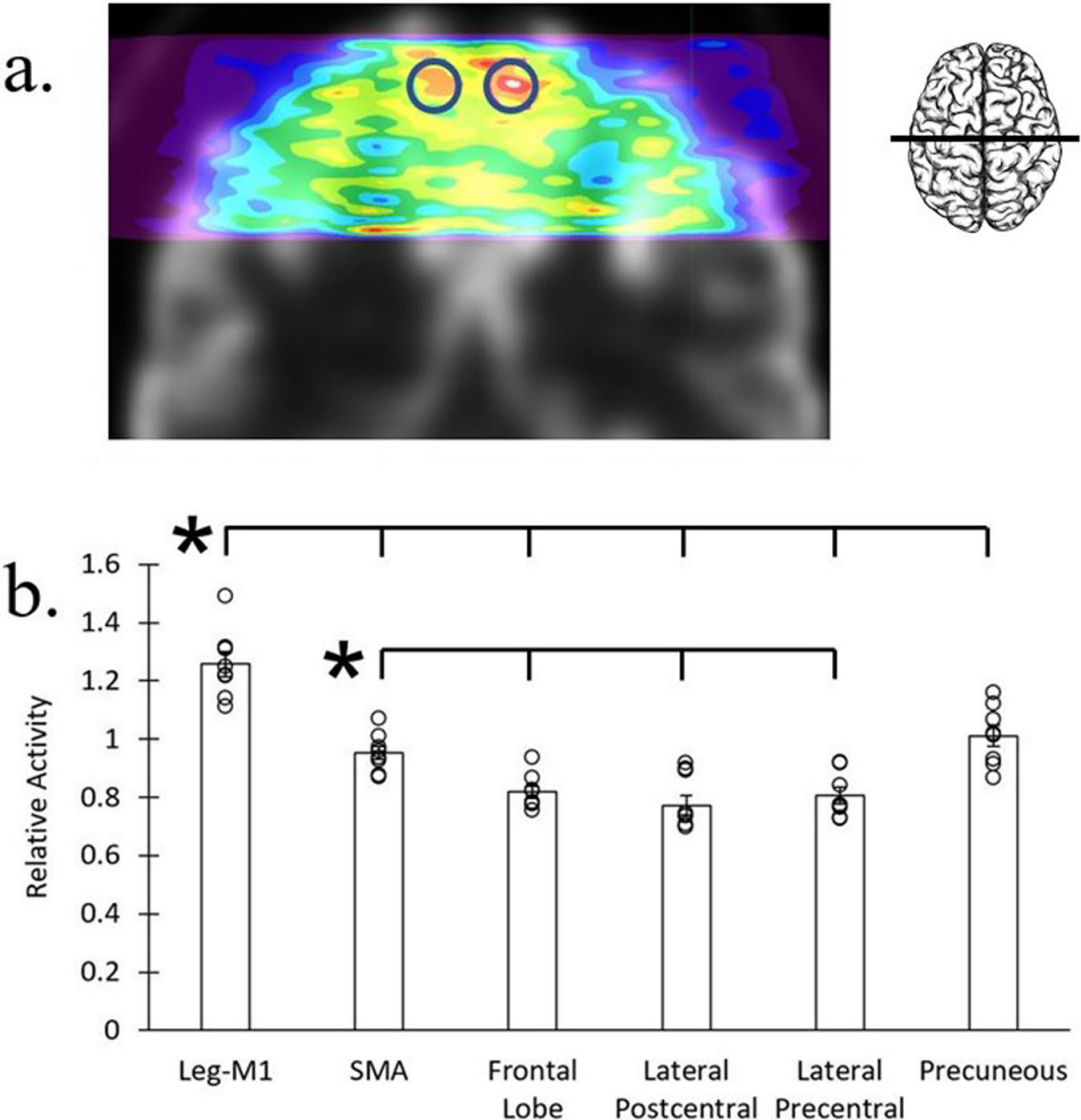
Ambulatory task-related activity. Walking-in-place versus standing-at-rest task-related activity (*n* = 8). **a** Motor task-related activity for an example participant on coronal slice through bilateral primary motor cortex. Blue circles indicate overlaid a priori leg motor cortex ROIs drawn from the central coordinates and size of an independent Delayed PET study of walking. Activity is represented by warm colors, red being the highest, and inset illustrates approximate slice location. **b** Average activity differences in a priori functional and anatomical cortical ROIs during the walking-in-place task period (*n* = 8). Relative activity in the bilateral leg-M1 ROI was significantly greater than all other ROIs (*p < 0.001, uncorrected). Error bars represent standard error. See Supplementary Table 1a for exact *p* values for all comparisons. Source data is in Supplementary Data 2.

Relative activity levels measured in a priori functional and anatomical ROIs of motor-related and non-motor brain regions were used to assess task-related activity over the 6 min activity period (the alternating 30 s of rest could not be analyzed separately, as this was not a bolus-infusion task and the FDG ligand would be sequestered, making it impossible to resolve walking-in-place vs. standing-at-rest activity in that timescale, see Supplementary Fig. 1). Data were normalized by dividing activity levels of a non-task involved frontal cortex reference ROI that could be measured in all participants (see Methods). Due to one under-dosed patient-participant and two in whom the leg-M1 ROIs were not adequately covered in the FOV placement, 8 participants were included in this analysis (Fig. 2). With imaging after a bolus injection being most similar to a previous functional Delayed-PET approach^41^, we found activity in that study’s bilateral leg-M1 ROI was significantly greater than activity in all other covered ROIs (Fig. 2a), as expected. Leg-M1 ROI activity versus mean activity of the whole brain covered within the FOV was elevated approximately 17.5% (*p* = 0.00000062). The activity in leg motor cortex was also increased relative to other individual ROIs; including vs. SMA, precentral gyrus, postcentral gyrus, precuneus, and frontal lobe (*p* < 0.001, Fig. 2b). Source data can be found in Supplementary Data 2, and for a list of exact *p* values for all comparisons, please see Supplementary Table 1.

Although we did not have a separate resting state session with AMPET, and so could not make a direct comparison of walking versus rest, we could examine the clinical PET brain scans to see if activity in leg motor and SMA was inherently elevated in this population sample. To do this, we applied the identical ROIs to each participant’s resting state scan derived from the three-minute clinical PET brain scan collected later the same day with 9–10 miCu FDG, and found no significant activity differences between leg motor cortex vs. any regions (see Methods and Supplementary Fig. 2, precuneus was elevated vs. post-central gyrus which is in line with standard elevation of that region during resting states^44^). This affirms that there was no inherently high levels of activity in the a priori selected leg-motor ROIs at rest for these participants, making it more likely that the elevated leg-M1 activity during our AMPET scan was related to the walking-in-place task, a validation of measure #1 and #2. Exact *p* values for clinical PET data are in Supplementary Table 1c.

Next, motor planning region, bilateral SMA, had significantly greater activation relative to other a priori control cortical regions not traditionally involved in motor execution, including the frontal lobe, the postcentral gyrus and precentral gyrus (*p* < 0.01), but significantly less activity than the bilateral leg-M1 ROI with *p* = 000019 (Leg M1 ROIs > SMA and Precuneus > Non-leg motor regions). The Precuneus ROI, which is also not traditionally considered a motor execution region, showed activation comparable to the SMA. This activation may have been related to the task-instruction of maintaining visual fixation on a computer screen and watching for instruction^44^. Overall, these outcomes supported our Validation measure #2 by revealing differential measures of activity in various predefined motor- versus non-motor-related cortical regions.

### Case study of a leg amputee patient

**Fig. 3:**
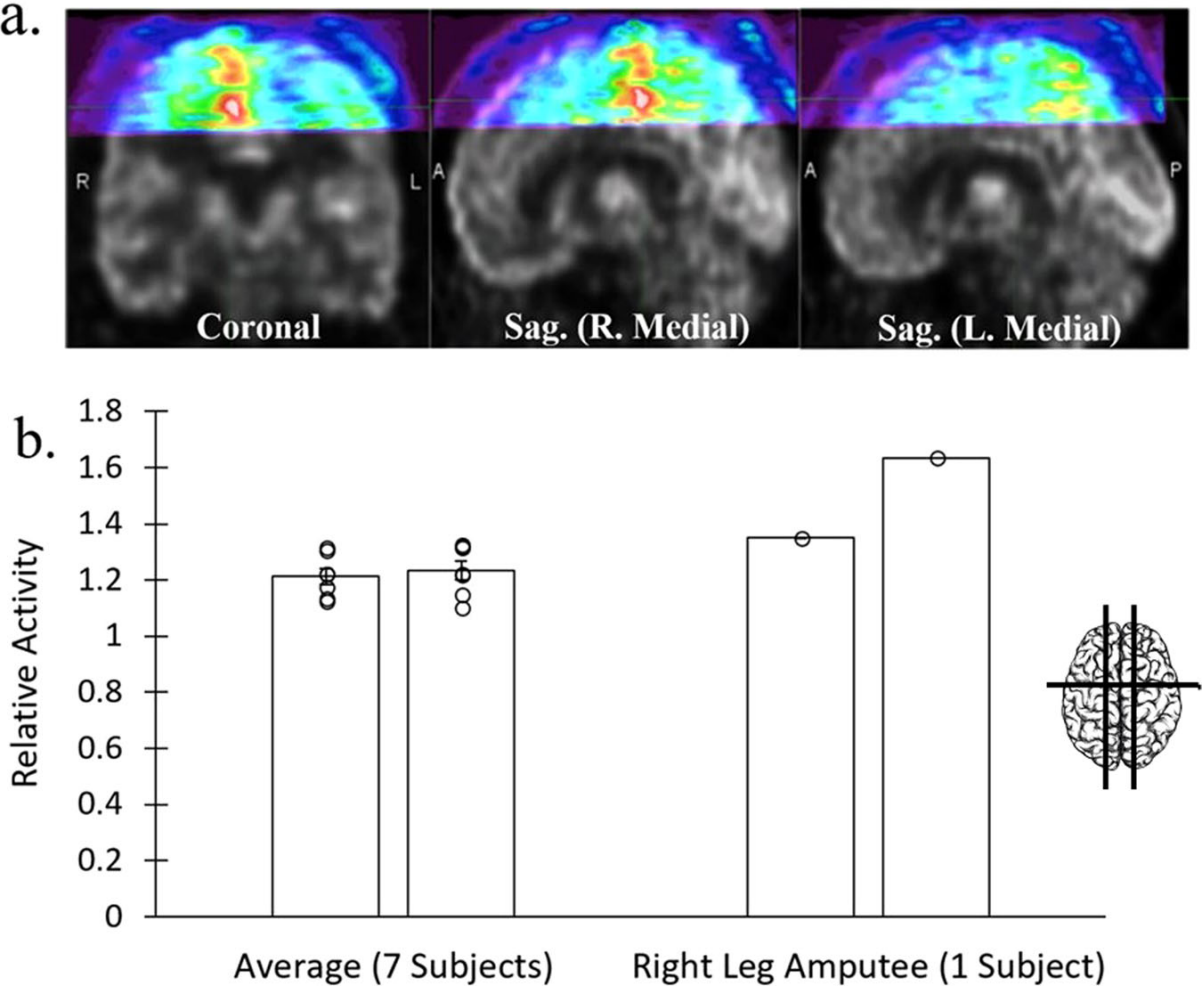
Unilateral dominance in amputee case study patient. Data from one participant with whole right leg amputation who performed the walking-in-place task while using their right prosthetic leg (*n* = 1), compared with the group data (*n* = 7). **a** AMPET images revealing greater activity (warm colors) in the right versus left hemisphere leg-M1 cortical regions, viewed in coronal slice and in right versus left parasagittal slices equidistant from midline. Right motor cortex represents the existing left leg. Brain inset shows approximate slice locations. **b** Average activity in a priori left (L) versus right (R) leg-M1 ROIs in group data (7 participants; mean ± SE relative activity) relative to the right-leg amputee participant (mean). Insets illustrates approximate slice locations. Error bars represent standard error. Source data is in Supplementary Data 3.

We were grateful to be able to image the brain of one patient-participant who had a full hip-to-foot prosthetic right leg (without articulating joints), who was able to perform the upright walking-in-place versus standing-at-rest block paradigm task, to contrast their activation with the remaining participants in Fig. 3. The walking motion of this participant involved stepping with their existing left leg, alternating with shifting their center-of-mass onto the prosthetic right leg. As might be predicted form amputee studies, brain FDG patterns showed greater metabolic activity in the right hemisphere leg-M1 ROI, representing the intact (contralateral) leg (Fig. 3a)^45–47^. In contrast, for the participants with both legs (*n* = 7), no significant activity difference was measured when comparing left versus right hemisphere leg-M1 ROIs, *p* = 0.37 (Fig. 3b, Source data in Supplementary Data 3). Although a larger sample of amputee patients would be needed to reach neuroscientific conclusions, this finding was consistent with the idea that movement of the intact left leg and joint musculature recruited greater activation of the representative brain region for coordinating walking, consistent with some animal studies (see Discussion).

### Additional imaging of deep brain and M1 regions

After the initial 6 min post-injection walking task period, the AMPET scanner was repositioned into one of two configurations with data being collected during minutes 11–16 post-injection. To ensure continual uptake, participants continued to perform a seated-walking versus seated-rest task. For this imaging period, half of the participants had been randomly selected to continue with collecting data from the top portions of the head. This M1 condition served as a within-group cross-validation measure that assessed the ability to consistently position the AMPET helmet. For the deep-brain condition, the other half of participants had the AMPET helmet positioned lower around the head to image deep lying brain structures that were initially out of range during the earlier portion of the experimental session. Each of these conditions is described below.

### Subset of participants with deep brain regions imaged

**Fig. 4:**
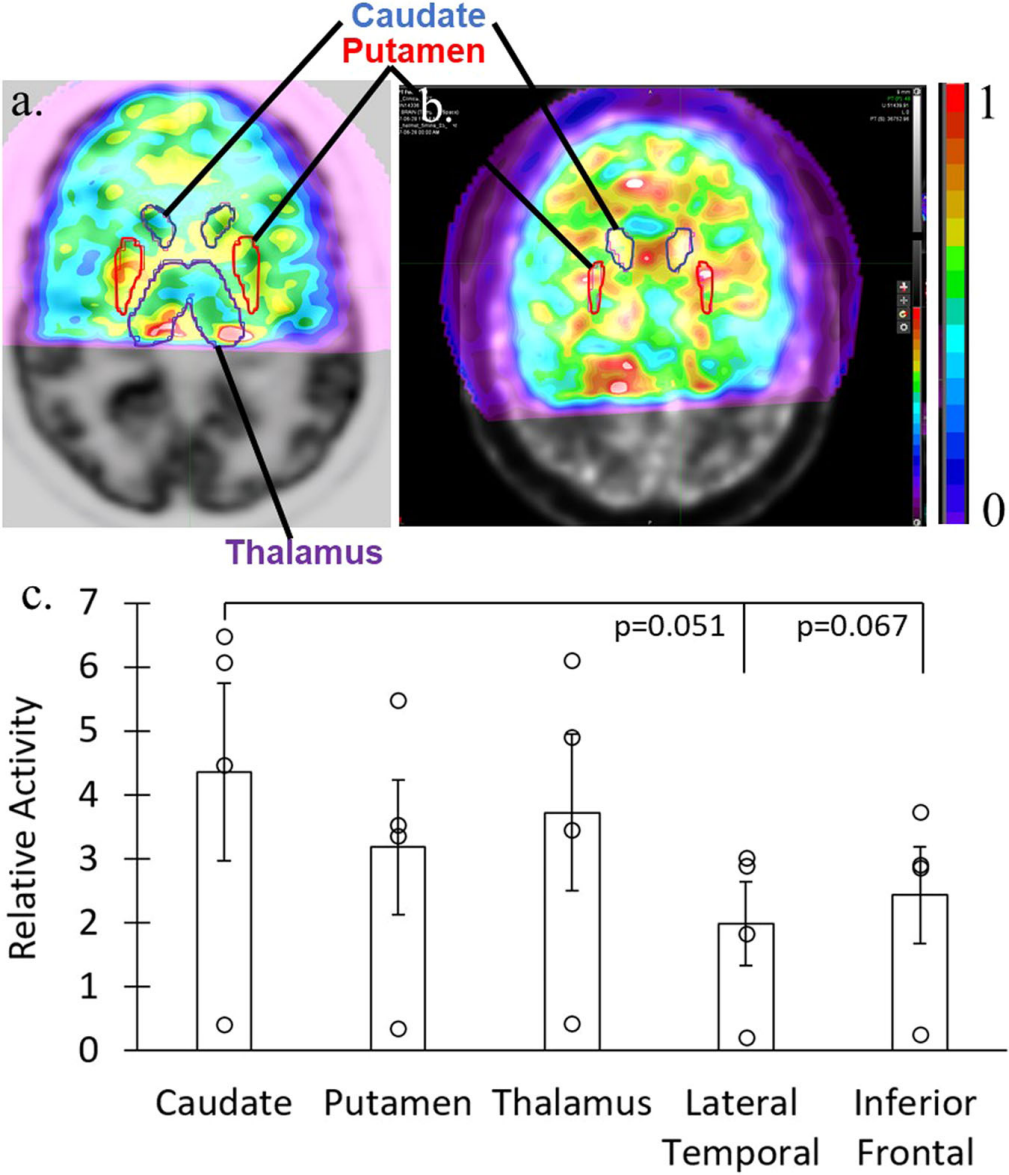
Deep brain activation in participant subset. Deep brain activity measures during the seated-walking versus seated-rest task, after repositioning the AMPET helmet lower around the head for a subset (*n* = 4) of the participants. **a**, **b** Activity in two example participants’ axial slices showing activity in deep brain regions, including overlaid a priori anatomical ROIs of the caudate, putamen and thalamus. **c** Charts showing average (mean ± SE) activity in various deep brain versus cortical ROIs. Error bars represent standard error. Source data is in Supplementary Data 4. See Supplementary Table 1b for exact *p* values for all comparisons.

For 5 participants we aimed to capture activity measures from deep lying brain structures. Four had coverage within predefined MIM database anatomical ROIs. One participant had helmet placement that was outside the intended region of coverage (again highlighting a need for improved helmet placement methods; see Discussion). We observed differential activation in deep brain structures, including the basal nuclei (Fig. 4a, b). For instance, this included a trend (Fig. 4c) for greater activation in the Caudate ROI versus Lateral Temporal cortex ROI (*p* = 0.051), and the Caudate ROI versus Inferior Frontal cortex ROI (*p* = 0.066). Although the results in this small subset only revealed a trend for differential activation, these outcomes nonetheless lend support towards affirming Validation measure #3, by revealing activation from various deep brain structures, here notably including the basal nuclei (caudate and putamen, Data in Supplementary Data 4, exact *p* values in Supplementary Table 1b).

### Subset of participants with leg-M1 ROI imaged again

At the later 11–16 min post-injection time window of data collection, activation in the bilateral leg-M1 ROIs (*n* = 5, randomly assigned for this re-positioning) was found to be significantly greater than the mean activity in the reference ROI (frontal lobe, *p* = 0.00089; Data in Supplementary Data 5) indicating a stable pattern that can be detected either in motion or with delayed imaging post-uptake of a sequestered ligand like FDG. Thus, this strategy served to cross-validate outcomes from later versus earlier stages of the AMPET scanning session, thereby further addressing Validation measure #2.

## Discussion

The present study, which sought to validate measures of brain imaging feasibility using our AMPET prototype, supports the potential for a developed imager to conduct ecologically-valid functional PET brain imaging during fully upright ambulatory motor tasks. Despite the limitations of our simplified qualitative method of imaging after a single bolus delivery (without arterial blood sampling) in a non-clinical neuropsychology laboratory (albeit radiation safety approved), and with a low ligand dose allotted in this convenience patient-participant sample, the functional data met our validation measures and we feel this achievement will warrant future study with a fully developed imager. To our knowledge, the robust, natural walking-related movements produced by our motor tasks have never before been tolerated or fully accommodated by any human brain imager that had a potential for deep brain, and eventually whole-brain, ecologically-valid imaging.

First, in testing for motion artifact, continuous imaging during a block-design motion task which alternated walking-in-place versus stillness, allowed us to test for any movement-related misalignment of the imager. Analyses show that even a simple friction fit helmet system limited brain-imager movement to less than a couple millimeters during this robust motor task.

In terms of functional brain imaging, we further demonstrate task-related activity in appropriate locomotion-task related brain regions in primary and supplementary motor cortices, including trend-level activity in deep lying basal nuclei (ganglia), in a small subset of participants with this region included in the FOV. We demonstrate a pattern of walking movement-related brain region activity, including activation similar to cortical findings by fNIRs studies of human ambulation^48^, with fNIRS being restricted to superficial brain (cortical only) regions. The imaging with our AMPET prototype is further congruent with results from the Delayed-PET walking study from which our a priori leg-M1 ROIs were derived^41^. However, in contrast to earlier fNIRS and PET studies^41,48^, the present study reveals a potentially more accurate relative measure of neuronal activation of the SMA (a moderately deep cortical region) versus primary motor cortex. Activation patterns in the present AMPET study were not confounded by the depth coverage issues inherent to fNIRS, or the inability to move about in an upright walking position with fMRI. Additionally, our small study subset indicates feasibility of deep brain imaging, including the basal nuclei, which have no coverage or highly compromised coverage with the surface imagers. Importantly, the ability to image basal nuclei and other deep brain structures such as the amygdala, hippocampus, and thalamus, is critically important for further understanding and assessing the progression(s) of multiple pathologies^41^ through the use of real-world behavioral tasks associated with movement disorders^25^.

Also, to our knowledge, there have been no other functional neuroimaging studies of leg motor cortex changes in human leg amputees. A study in monkeys suggests structural atrophy may be expected in the motor cortex representing the absent leg^45,47^. The motor cortex differences observed in the present AMPET study may thus reflect reduced activity/cortical atrophy in cortex contralateral to (representing) the amputated leg, or that it may take more neural effort to control the harder working existing leg to compensate the missing limb. Confirming and differentiating these preliminary findings would require further study with arterial blood sampling and ideally with repeated measures as a longitudinal study, but is an example of the types of studies that would be feasible with the advances afforded by a fully-developed AMPET helmet system.

The above AMPET prototype demonstrations indicate that expected results as well as unexpected insights may be revealed as a result of using paradigms that allow natural behaviors in upright positions. Such insights could expand neuroimaging research such as assessing the neural mechanisms of skill acquisition of robust motor task performance^49^, or engaging in tasks involving balance or motor coordination, which are skills impaired in a wide variety of brain injuries and diseases^50^. Many such tasks are critically affected by deep lying brain function and cannot be performed while seated or lying down. In addition, one could also adapt the AMPET prototype with a body harness system for those at fall risk^51,52^.

Beyond motor research, a developed AMPET-like imager would enable other types of ecologically-valid behavioral studies. Such may include accommodating tasks involving social interactions that require upright motion, such as leaning towards or away from a person to indicate friendliness/fear/dominance, vocalizations with corresponding communicative postures and gestures, use of objects such as tools and surgical implements, playing musical instruments/singing, and accommodating behavioral responses with affective motor components such as laughter, disgust/fear, recoil and surprise^13^. We also envision the AMPET imager to be integrated with the growing field of virtual reality (VR technologies), which has a beneficial trade-off between controlled variables and natural behavior^53,54^. Working with radiation safety personnel, if a public space or outdoor area could be temporarily restricted, human behavior in natural settings could be studied, such as nature or art immersion environments^55^.

The AMPET prototype may also allow one to image, without sedation, patient populations who cannot remain still or lie flat comfortably or safely due to a variety of issues including age (too young or elderly), intoxication/cognitive impairment (difficulty following instructions), movement disorders (e.g., Tourette’s Syndrome, Parkinson’s Disease), or stroke risk (greater ischemic risk when lying supine). Such a device could enable research and diagnostic imaging in these populations, serving as an imperative alternative to traditional sedation-requiring MRI or PET.

An overarching aim of the present study, given our laboratory experiences using the device with human participants and the device system performance on the validation measures of feasibility, was to prioritize upgrades to the AMPET imager for this and other similar systems that may be under development. Based on our successes and challenges, priorities for development include adding an optical (e.g., infrared) positioning system, increasing axial FOV by redistributing the detector material to attain larger angular coverage (sparse structure bioengineering), and adding a lightweight gimbal mechanism to allow greater range of head motions and speeds.

We consider a positioning system to be the most simple and necessary upgrade, as some of our attempts to capture task-appropriate regions (motor cortices and/or deep brain structures) failed, due to incorrect visual placement around the head. A very lightweight system for maximum participant mobility may need to have a limited FOV (although we do expect that detector advances will increase FOV per se, see below). This is not unprecedented, as in the early years of fMRI research, the FOV was more limited, and the brain coverage for a given study needed to be carefully chosen to include the brain regions most relevant to a study’s hypotheses^56^. An infrared/optical system could be employed to utilize a limited axial FOV PET image^57^, similar to that used in other modalities like transcranial magnetic stimulation (TMS), in which one can show the real-time position of the TMS wand trigger zone relative to the participants’ 3D brain rendering^58,59^. In addition, such systems could track the imager relative to the brain to provide input to improve image quality via motion tracking residue error correction^60^. Although an average brain and skull could be used for alignment relative to anatomical landmarks (nasion, preauricular points, etc.), for best anatomical placement accuracy and for post-processing attenuation correction, it is ideal to use a participant’s own brain, collected via anatomical MRI series (T1, T2, FLAIR) or CT scan^61^.

In terms of upgrade suggestions for expanding FOV, based on simulation studies, brain coverage could be increased considerably, and the imager could still remain lightweight with a wider field-of-view (FOV), using thinner scintillators as a potential trade-off^38^. The thickness of the crystal affects the stopping power of the annihilation photons, and thus the number of coincident events detected, which is an important factor in the overall sensitivity of the device. The vertical length of the cylinder also plays a critical role, so optimization of the scanner could be performed by rebalancing these parameters. Our team has previously published results that modeled the number and placement of (and angular coverage by) detectors as well as different crystal thicknesses, time-of-flight (TOF) parameters, and the tradeoffs that occur therein^57^. Ours and other simulations indicate that the advantages of greater volume coverage exceed the advantages of locally thicker crystal^38^ due to PET-specific capture physics. Extended angular coverage would also facilitate non-invasive carotid arterial sampling to enable quantitative dynamic imaging in place of arterial blood draws^62,63^.

In terms of upgrade suggestions for motion-enabling support, this study of our AMPET prototype and its earlier Helmet_PET prototypes together demonstrate that a simple robust, and safe support system may be all that is needed for use of relatively lightweight PET brain imagers^40^ for some applications. A mock-imager study from our group showed tolerance of these same motions with a larger weighted faux device (~10 kg), supported using a smooth counterweight system that was built onto an adapted physical therapy device called the Biodex Unweighing system^52^. This commercial support system additionally has a harness that supports a patient’s body weight to an adjustable degree (the Unweighing system was originally designed for physical therapy applications, for example while patients re-learn to walk after a stroke^52^). With a full-room infrared (IR) tracking setup, a weighted mock-up imager supported from a single support point above the head shows no significant movement of the imager relative to the head during rotation, upright walking in-place, walking on treadmill, and walking while pushing the wheeled support frame. However, the IR sensor study shows significant movement of  the imager relative to the head when human participants engage in movements like nodding (pitch) and moving ear towards shoulder (yaw), in the sagittal and frontal planes^51,52^. Such movement limitations from a single source support should not affect walking and many other upright tasks, but if full movement needed, one could use a gyroscope mechanism^51,52^ similar to the gimbal mechanism used in the RatCap^29^.

We further envision support systems incorporating robotics that would allow for substantially heavier imagers, affording very high sensitivity and/or whole-brain FOV^64^, although safety and accommodating momentum would require careful planning and testing. Alternatively, in order to take advantage of freedom from any tethers, one could utilize a backpack support^65,66^, although the heaviness of such a system currently being developed, likened to wearing a large motorcycle helmet and backpack, may restrict researchers to relatively strong participants^38^.

Lastly, for neuroscientists who are unfamiliar with PET, who may be interested in utilizing such a motion-enabling PET system, certain logistics specific to PET, such as use of radioligands, would require educational familiarization for researchers and for human participant IRBs. Neuroscience laboratory spaces could take advantage of either temporary or permanent radiation area designations and multiple flood scans at different temperatures could ensure the best match for the scanning conditions.

In terms of advantages for neuroscience research, combining the quantitative and/or neurotransmitter-specific power of PET with motion-tolerant imaging properties could advance study of state- and task-related brain activation related to uniquely human activities. Task periods could be longer than those allowed by fMRI or fNIRS, due to physiology and the physics of blood flow-related imaging markers^67^. This is important in tasks that cannot be easily turned on and off, such as meditation: An earlier study of meditation had to limit meditation cycles to unnaturally short periods, as well as compare to an unideal baseline of ‘zoning-out’^68^ due to the qualitative, relative-to-baseline nature of blood flow imaging in block paradigms. Upright, motion-enabling PET with quantitative methods would thus be applicable to the studies of longer duration, upright, behavioral tasks incorporating mental health therapies, elite athletic training, art, music, flow-states, pharmaceuticals, and substances of abuse.

The small footprint of our imager, which, unlike MEG^19^, does not require a special shielded room, could make it a complementary modality. For example, laboratories already using motion-enabling fNIRS or HD-DOT may benefit from the deeper brain coverage or the ability to look at other markers, such as radiolabeled neurotransmitters. The range of radioligands available, including markers of oxygen use, inflammation, endogenous production of neurotransmitters, and gene regulation with histone deacetylase (HDAC)^69–72^, may be critically informative in the context of active behavioral tasks. Such opportunities could also enable a more accurate avenue of complementary animal and human behavioral studies, such as in addiction-related behaviors^73^. Overall, an AMPET imager with the deep brain coverage, timing advances, and ligand options of PET, combined with motion-tolerance, upright posture, and small footprint, could expand the reach of multiple neuroscience fields.

### Supplementary information

Supplementary Information

Description of Additional Supplementary Files

Supplementary Data 1

Supplementary Data 2

Supplementary Data 3

Supplementary Data 4

Supplementary Data 5

Supplementary Data 6

Supplementary Data 7

Reporting Summary

Cover Art

## Data Availability

The source data for each average ROI used in figures are provided in Supplementary Data 1 for Fig. 1, Supplementary Data 2 for Fig. 2, Supplementary Data 3 for Fig. 3, Supplementary Data 4 for Fig. 4, Supplementary Data 5 for section on leg-motor ROI repositioning, Supplementary Data 6 for Supplementary Fig. 1 and Supplementary Data 7 for Supplementary Fig. 2. The raw data analyzed in the current study are not publicly available due to data protection issues but are available from the corresponding author on reasonable request, such as if one wants to collaborate or to directly compare with a similar ambulatory PET system.

## Code availability

The custom software is proprietary to WVU. It was not developed for this project^42^ and was used as a courtesy of Dr. Ray Raylman (WVU researchers and potential collaborators may request Dr. Raylman), with adaptations specific to the hardware of the unique imager. There are analogous freeware packages, such as STIR (https://stir.sourceforge.net/), that would be able to reconstruct the data of this format.
