# Supplementary material for: Real-time motion-enabling positron emission tomography of the brain of upright ambulatory humans
Source: Commun Med (Lond). 2024 Jun 13;4:117. doi: 10.1038/s43856-024-00547-2 (PMC11176317; doi:10.1038/s43856-024-00547-2)
Supplement: Supplementary file 1 — Supplementary Information [file 43856_2024_547_MOESM1_ESM.pdf]

SUPPLEMENTARY INFORMATION

Real-time motion-enabling positron emission tomography of the brain of upright ambulatory humans

Nanda K Siva[1] (Co-First Author), Chris Bauer[2] (Co-First Author), Colson Glover[1], Alexander Stolin[1], Sonia Chandi[1], Helen Melnick[1], Gary Marano[1], Ben Parker[1], Mary Beth Mandich[1], James W Lewis[1], Jinyi Qi[3], Si Gao[1], Kaylee Nott[1], Stan Majewski[3], and Julie A. Brefczynski-Lewis[1]

[1]West Virginia University, Morgantown, WV, USA
[2]University of Kentucky, College of Medicine, Lexington, KY, USA
[3]University of California – Davis, Department of Biomedical Engineering, Davis, CA, USA

Key words: positron emission tomography (PET), mobile PET, deep brain imaging, Helmet_PET, Amputee cortical plasticity mapping, basal ganglia

Correspondence should be addressed to:
Julie A. Brefczynski-Lewis, PhD.
Department of Neuroscience
P.O. Box 9303
West Virginia University
Morgantown, WV 26506
Phone: 304 293-4820
Fax: 304-293-3850
Email: jblewis@hsc.wvu.edu
4 Figures (3 color)

Supplementary Figures.

Supplementary Figure 1. Example of absolute activity measures over time. Graph illustrates sequestering of F18-FDG during uptake, plotting mean ROI absolute activity every 30 seconds, as measured by the AMPET prototype for an example subject. Lines show initial rapid uptake at 30 seconds post injection and then a slower uptake over time for the bilateral leg motor ROI (dashed black) and neutral frontal ROI (solid grey). Source data is in **Supplementary Data 6**.

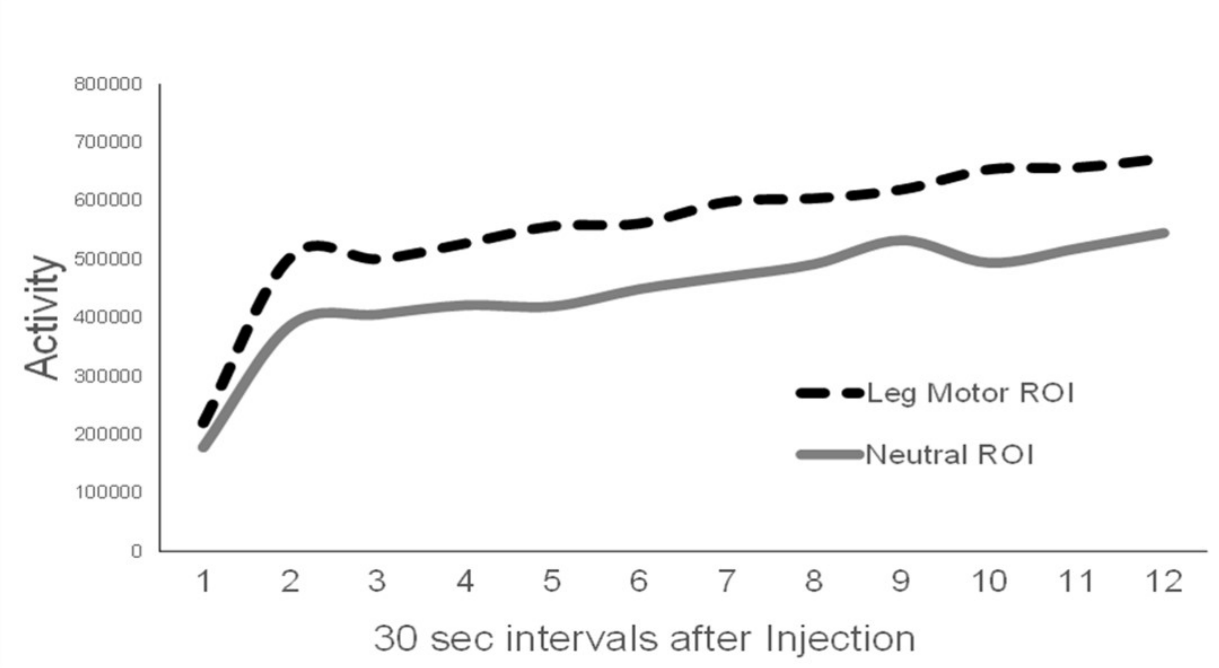

Supplementary Figure 2. Clinical PET resting scan. Relative activity measures from the clinical PET scan within the predefined bilateral ROIs used for the AMPET data analysis (n=8, same 8 participants as Fig. 2 for comparison). No significant differences observed suggest that there were no inhomogeneities of relative uptake specific to the motor regions. Error bars represent standard error. Source data is in **Supplementary Data 7**. See **Supplementary Table 1c** for exact p-values for all comparisons.

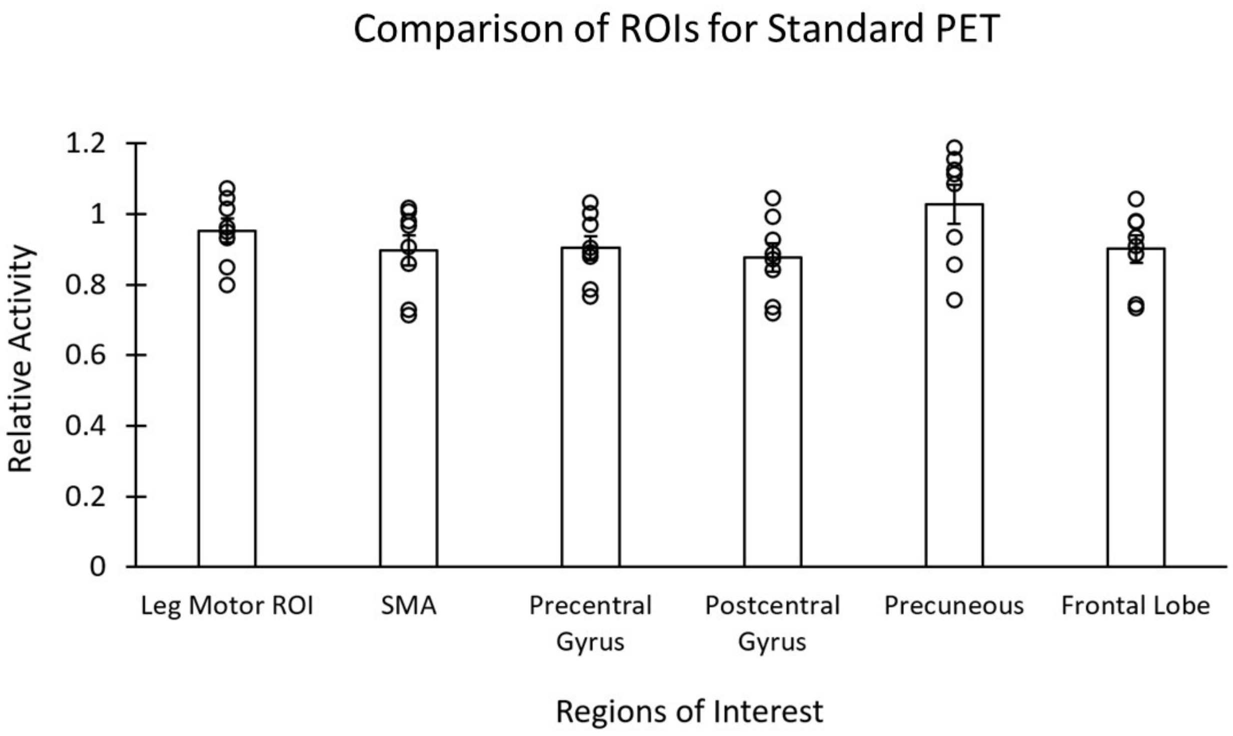

Supplementary Table.

Supplementary Table 1. Exact p-values. Exact p-values for all comparisons in a. Figure 1, b. Figure 4 and c. Supplementary Figure 2. All other exact p-values are included in the text.

a. Exact p-values. Exact p-values for Figure 2, Ambulatory task-related activity.

	Leg_M1	SMA	Frontal Lobe	Lateral Postcentral	Lateral Precentral	Precuneous
Leg_M1	-	0.000019	0.00000021	0.00000051	0.00000036	0.00059
SMA	-	-	0.00078	0.0014	0.0013	0.193
Frontal Lobe	-	-	-	0.503	0.729	0.0004
Lateral Postcentral	-	-	-	-	0.74	0.00052
Lateral Precentral	-	-	-	-	-	0.0005
Precuneous	-	-	-	-	-	-

b. Exact p-values for Figure 4, Deep brain activation in data subset.

	Caudate	Putamen	Thalamus	Lateral Temporal	Inferior Frontal
Caudate	-	0.123	0.107	0.051	0.067
Putamen	-	-	0.234	0.133	0.126
Thalamus	-	-	-	0.066	0.098
Lateral Temporal	-	-	-	-	0.231
Inferior Frontal	-	-	-	-	-

c. Exact p-values for Supplementary Figure 2, Clinical PET Resting Scan.

	Leg_M1	SMA	Precentral Gyrus	PostCentral Gyrus	Precuneous	Frontal Lobe
Leg_M1	-	0.322	0.319	0.169	0.277	0.327
SMA	-	-	0.908	0.735	0.087	0.957
Precentral Gyrus	-	-	-	0.621	0.081	0.951
PostCentral Gyrus	-	-	-	-	0.048	0.683
Precuneous	-	-	-	-	-	0.086
Frontal Lobe	-	-	-	-	-	-

Supplementary Data Legends.

Supplementary Data 1. Individual participant data for Figure 1. ROI average amplitude during active Walking-in-place versus Standing-at-rest periods.

Supplementary Data 2. Individual participant data for Figure 2. Data are mean normalized data for each ROI (n=8) for the walking-in-place task.

Supplementary Data 3. Individual Data for Figure 3. Table contains individual mean normalized activity for the Amputee Participant, as well as the other 7 participants for the left and right *a priori* leg ROIs.

Supplementary Data 4. Individual participant data for Figure 4. Mean relative activity in deep brain regions for the four participants that had mid-brain coverage in the seated walking task. (n=4).

Supplementary Data 5. Individual participant data for seated walking with M1 coverage. Mean relative activity in M1 vs Frontal Lobe reference region in the seated walking task. (n=5) The final three listed participants' frontal lobe ROI coverage was not fully complete.

Supplementary Data 6. Uptake curves. Raw activity measures taken every 30 seconds for an example participant of F18-FDG uptake during the walking and standing in place period.

Supplementary Data 7. Clinical PET ROI data. Relative activity measures from the clinical PET scan with the predefined bilateral ROIs used for the AMPET data analysis.
