# Supplementary material for: Real-time motion-enabling positron emission tomography of the brain of upright ambulatory humans
Source: Commun Med (Lond). 2024 Jun 13;4:117. doi: 10.1038/s43856-024-00547-2 (PMC11176317; doi:10.1038/s43856-024-00547-2)
Supplement: Supplementary file 2 — Description of Additional Supplementary Files [file 43856_2024_547_MOESM2_ESM.pdf]

Description of Additional Supplementary Files

File name: Supplementary data 1
Description: Individual participant data for Figure 1. ROI average amplitude during active Walking-in-place versus Standing-at-rest periods.

File name: Supplementary data 2
Description: Individual participant data for Figure 2. Data are mean normalized data for each ROI (n=8) for the walking-in-place task.

File name: Supplementary data 3
Description: Individual Data for Figure 3. Table contains individual mean normalized activity for the Amputee Participant, as well as the other 7 participants for the left and right a priori leg ROIs.

File name: Supplementary data 4
Description: Individual participant data for Figure 4. Mean relative activity in deep brain regions for the four participants that had mid-brain coverage in the seated walking task. (n=4).

File name: Supplementary data 5
Description: Individual participant data for repositioning data with M1 coverage. Mean relative activity in M1 vs Frontal Lobe reference region in the seated walking task. (n=5) The final three listed participants' frontal lobe ROI coverage was not fully complete.

File name: Supplementary data 6
Description: Uptake curves. Raw activity measures taken every 30 seconds for an example participant of F18-FDG uptake during the walking and standing in place period.

File name: Supplementary data 7
Description: Clinical PET ROI data. Relative activity measures from the clinical PET scan with the predefined bilateral ROIs used for the AMPET data analysis.
