# Supplementary material for: Real-time motion-enabling positron emission tomography of the brain of upright ambulatory humans
Source: Commun Med (Lond). 2024 Jun 13;4:117. doi: 10.1038/s43856-024-00547-2 (PMC11176317; doi:10.1038/s43856-024-00547-2)
Supplement: Supplementary file 10 — Reporting Summary [file 43856_2024_547_MOESM10_ESM.pdf]

nature portfolio

Corresponding author(s): Julie Brefczynski-Lewis

Last updated by author(s): May 13, 2024

Reporting Summary

Nature Portfolio wishes to improve the reproducibility of the work that we publish. This form provides structure for consistency and transparency in reporting. For further information on Nature Portfolio policies, see our Editorial Policies and the Editorial Policy Checklist.

Statistics

For all statistical analyses, confirm that the following items are present in the figure legend, table legend, main text, or Methods section.

n/a	Confirmed	
☐	☒	The exact sample size (n) for each experimental group/condition, given as a discrete number and unit of measurement
☐	☒	A statement on whether measurements were taken from distinct samples or whether the same sample was measured repeatedly
☐	☒	The statistical test(s) used AND whether they are one- or two-sided<br>*Only common tests should be described solely by name; describe more complex techniques in the Methods section.*
☒	☐	A description of all covariates tested
☐	☒	A description of any assumptions or corrections, such as tests of normality and adjustment for multiple comparisons
☐	☒	A full description of the statistical parameters including central tendency (e.g. means) or other basic estimates (e.g. regression coefficient) AND variation (e.g. standard deviation) or associated estimates of uncertainty (e.g. confidence intervals)
☐	☒	For null hypothesis testing, the test statistic (e.g. F, t, r) with confidence intervals, effect sizes, degrees of freedom and P value noted<br>*Give P values as exact values whenever suitable.*
☒	☐	For Bayesian analysis, information on the choice of priors and Markov chain Monte Carlo settings
☒	☐	For hierarchical and complex designs, identification of the appropriate level for tests and full reporting of outcomes
☐	☒	Estimates of effect sizes (e.g. Cohen's d, Pearson's r), indicating how they were calculated

Our web collection on statistics for biologists contains articles on many of the points above.

Software and code

Policy information about availability of computer code

Data collection	Custom-developed software specific to this imager, iiterative reconstruction software based on MLEM (Maximum-Likelihood Expectation-Maximization) algorithm, Smith, M. F., Raylman, R. R., Majewski, S. & Weisenberger, A. G. Positron emission mammography with tomographic acquisition using dual planar detectors: initial evaluations. Phys Med Biol 49, 2437-2452, doi:10.1088/0031-9155/49/11/022 (2004).
Data analysis	ImageJ research software (http://imagej.nih.gov/ij/) MIM software (version 6.6) and analyzed using MIMneuro and MIMfusion. First, the clinical PET scans of each participant were aligned to the CT scans automatically using the MIM BrainAlign algorithm (MIM Software, Inc., Cleveland, Ohio, USA), statistical software R (version 3.6.3, The R Foundation for Statistical Computing), JASP (version 0.8.1.1) and Microsoft Excel (version 14.6.5)

For manuscripts utilizing custom algorithms or software that are central to the research but not yet described in published literature, software must be made available to editors and reviewers. We strongly encourage code deposition in a community repository (e.g. GitHub). See the Nature Portfolio guidelines for submitting code & software for further information.

Data

Policy information about availability of data

All manuscripts must include a data availability statement. This statement should provide the following information, where applicable:

- Accession codes, unique identifiers, or web links for publicly available datasets
- A description of any restrictions on data availability
- For clinical datasets or third party data, please ensure that the statement adheres to our policy

Source data for each average ROI used in figures are provided with this paper (SI Tables 1 and 2). The data sets analyzed in the current study are not publicly available due to data protection issues but are available from the corresponding author on reasonable request.

Human research participants

Policy information about studies involving human research participants and Sex and Gender in Research.

Reporting on sex and gender	Biological sex was collected: 7 females and 4 males. We did not query on gender, as sex/gender differences were not relevant in this pilot demonstration of device function.
Population characteristics	Participants were all Caucasian of both sexes (7 females and 4 males) with a mean weight of 76.1 kilograms (167.8 pounds) and mean age of 53 years (age 25-66). One participant's data was omitted from the analysis due to an injection amount below the allotted range of 1-2 millicurie due to a timing delay, and two participants' trials were omitted from the walking task analysis, as placement of the scanner was below the leg motor ROI. This left a sample size of n=10 for the motion analysis and n=8 for the task analysis, which included a patient with a leg prosthetic. All participants were able to perform the limited locomotor tasks required in this study.
Recruitment	Patients scheduled for a clinical PET scan, without neurological disorders or brain-involved cancers, had the study described during the call in which they were scheduled for the clinical scan, since they would need to come in early for our study. They were also asked if they were ambulatory and able to walk. Self-selection or biasing is not applicable.
Ethics oversight	West Virginia University Office of Research Compliance.

Note that full information on the approval of the study protocol must also be provided in the manuscript.

Field-specific reporting

Please select the one below that is the best fit for your research. If you are not sure, read the appropriate sections before making your selection.

☒ Life sciences ☐ Behavioural & social sciences ☐ Ecological, evolutionary & environmental sciences

For a reference copy of the document with all sections, see nature.com/documents/nr-reporting-summary-flat.pdf

Life sciences study design

All studies must disclose on these points even when the disclosure is negative.

Sample size	The sample size was one of convenience in this first-in-human trial. The population was vulnerable (cancer patients coming in for a diagnostic PET scan) so we chose 10-12 subjects in our IRB as a minimum to get some meaningful results without being a burden. We recruited 11 participants, but one had a degraded radioactive ligand, and in several tests the region of interest was not captured in the field of view.
Data exclusions	Due to a delay in consent time/setting up the device, one subject was excluded as the FDG ligand that had been drawn had degraded below our minimal predetermined amount. For the walking task analyses, two participants' trials were omitted due to AMPET helmet placement that was inadvertently positioned below the leg motor Regions of Interest (ROIs). Thus, we had a sample size of n=10 for the motion tolerance analyses (validation measure #1), and a sample size of n=8 for the Walking-in-place task analyses (validation measure #2), and a subset of n=5 (of the 10) participants for re-placement of the AMPET to measure deep brain structure activity (validation measure #3).
Replication	n/a
Randomization	n/a
Blinding	n/a

Reporting for specific materials, systems and methods

We require information from authors about some types of materials, experimental systems and methods used in many studies. Here, indicate whether each material, system or method listed is relevant to your study. If you are not sure if a list item applies to your research, read the appropriate section before selecting a response.

Materials & experimental systems

n/a	Involved in the study
☒	☐ Antibodies
☒	☐ Eukaryotic cell lines
☒	☐ Palaeontology and archaeology
☒	☐ Animals and other organisms
☒	☐ Clinical data
☒	☐ Dual use research of concern

Methods

n/a	Involved in the study
☒	☐ ChIP-seq
☒	☐ Flow cytometry
☒	☐ MRI-based neuroimaging
