# Supplementary figures and images for: Real-time motion-enabling positron emission tomography of the brain of upright ambulatory humans
Source: Commun Med (Lond). 2024 Jun 13;4:117. doi: 10.1038/s43856-024-00547-2 (PMC11176317; doi:10.1038/s43856-024-00547-2)

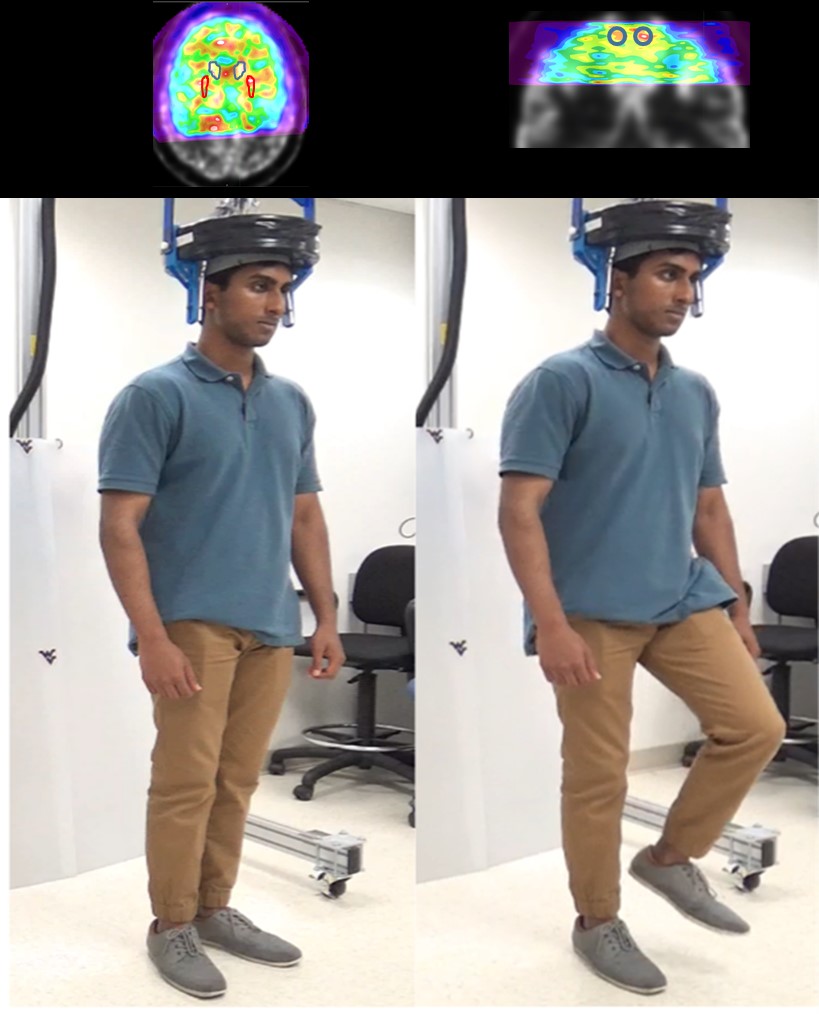

Supplement: Supplementary file 11 — Cover Art [file 43856_2024_547_MOESM11_ESM.jpg]
